# EXPERIENCES FROM A RCMAR SCIENTIST (ALUMNA): A MEANINGFUL AND REWARDING JOURNEY

**DOI:** 10.1093/geroni/igae098.0683

**Published:** 2024-12-31

**Authors:** Tamara Baker

**Affiliations:** University of North Carolina, Chapel Hill, Chapel Hill, North Carolina, United States

## Abstract

RCMAR was established to develop a research and mentoring infrastructure that fosters rigorous behavioral and social science research on aging in a high priority research area that can advance aging-relevant scientific discoveries, lead to the elimination of health disparities and health inequities, and improve the health and well-being of older adults. The primary objectives of RCMAR are 1) career development, which aims to support the career development of early-career scientists conducting social, behavioral, psychological, and economic research related to aging, and 2) advancement of scientific discoveries related to aging. Scientists participating in this program are afforded opportunities to establish a successful program of research; personalized guidance through professional development activities, helping scientists develop skills, knowledge, and confidence in the field; networking opportunities, expanding their professional network and potential career opportunities; personal growth; feedback and advice, helping scientists improve performance and make informed decisions; career advancement, receiving guidance on strategies for success; and knowledge transfer, fostering continuity and innovation in the field. These opportunities are recognized in developing some of the most established scholars in the discipline. Dr. Baker, a former RCMAR scientist will reflect on how her participation as a RCMAR scientist has allowed her to build a successful research career on the intersection of social determinants and chronic pain in older adults; be an advocate in diversifying the workforce; and become a leader in the gerontology discipline. Highlighting these successes is intended to broaden the outreach of program while encouraging others to consider this opportunity.

